# Hemodynamics of self-expanding versus balloon-expandable transcatheter heart valves in relation to native aortic annulus anatomy

**DOI:** 10.1007/s00392-022-02046-7

**Published:** 2022-06-15

**Authors:** Sarah Schmidt, Vera Fortmeier, Sebastian Ludwig, Hendrik Wienemann, Maria Isabel Körber, Samuel Lee, Max Meertens, Sascha Macherey, Christos Iliadis, Elmar Kuhn, Kaveh Eghbalzadeh, Sabine Bleiziffer, Stephan Baldus, Niklas Schofer, Tanja Katharina Rudolph, Matti Adam, Victor Mauri

**Affiliations:** 1 Department of Cardiology, Heart Center, Faculty of Medicine, University of Cologne, Matti Adam Kerpener Str. 62, 50937 Cologne, Germany; 2 General and Interventional Cardiology, Heart and Diabetes Centre NRW, Bad Oeynhausen, Ruhr University Bochum, Bochum, Germany; 3 Department of Cardiology, University Heart and Vascular Center Hamburg, Hamburg, Germany; 4 Department of Cardiothoracic Surgery, Heart Centre, Faculty of Medicine, University of Cologne, Cologne, Germany; 5 Department of Cardiothoracic Surgery, Heart and Diabetes Centre NRW, Bad Oeynhausen, Ruhr University Bochum, Bochum, Germany

**Keywords:** SAPIEN 3, ACURATE neo, Evolut R, Evolut pro, TAVR

## Abstract

**Objectives:**

This study aimed to compare hemodynamic characteristics of different self-expanding (SE) and balloon-expandable (BE) transcatheter heart valves (THV) in relation to native aortic annulus anatomy.

**Background:**

A patient centered THV selection becomes increasingly important as indications for transcatheter aortic valve replacement (TAVR) are extended towards lower risk populations.

**Methods:**

Hemodynamic parameters including mean gradient (MG), effective orifice area (EOA), Doppler velocity index (DVI), degree of paravalvular regurgitation (PVR) and patient-prosthesis mismatch (PPM) were compared by valve type, label size and in relation to quintiles of native aortic annulus area.

**Results:**

2609 patients were treated at 3 centers in Germany with SAPIEN 3 (*n* = 1146), ACURATE Neo (*n* = 649), Evolut R (*n* = 546) or Evolut Pro (*n* = 268) THV. SE THVs provided superior hemodynamics in terms of larger EOA, higher DVI and lower MG compared to BE THV, especially in patients with small aortic annuli. Severe PPM was less frequent in SE treated patients. The rate of PVR ≥ moderate was comparable for SE and BE devices in smaller annular dimensions, but remarkably lower for BE TAVR in large aortic annular dimensions (> 547.64 mm^2^) (2% BE THV vs. > 10% for SE THV; *p* < 0.001).

**Conclusions:**

Patients with small aortic annular dimensions may benefit hemodynamically from SE THV. With increasing annulus size, BE THV may have advantages since PVR ≥ moderate occurs less frequently.

**Graphical abstract:**

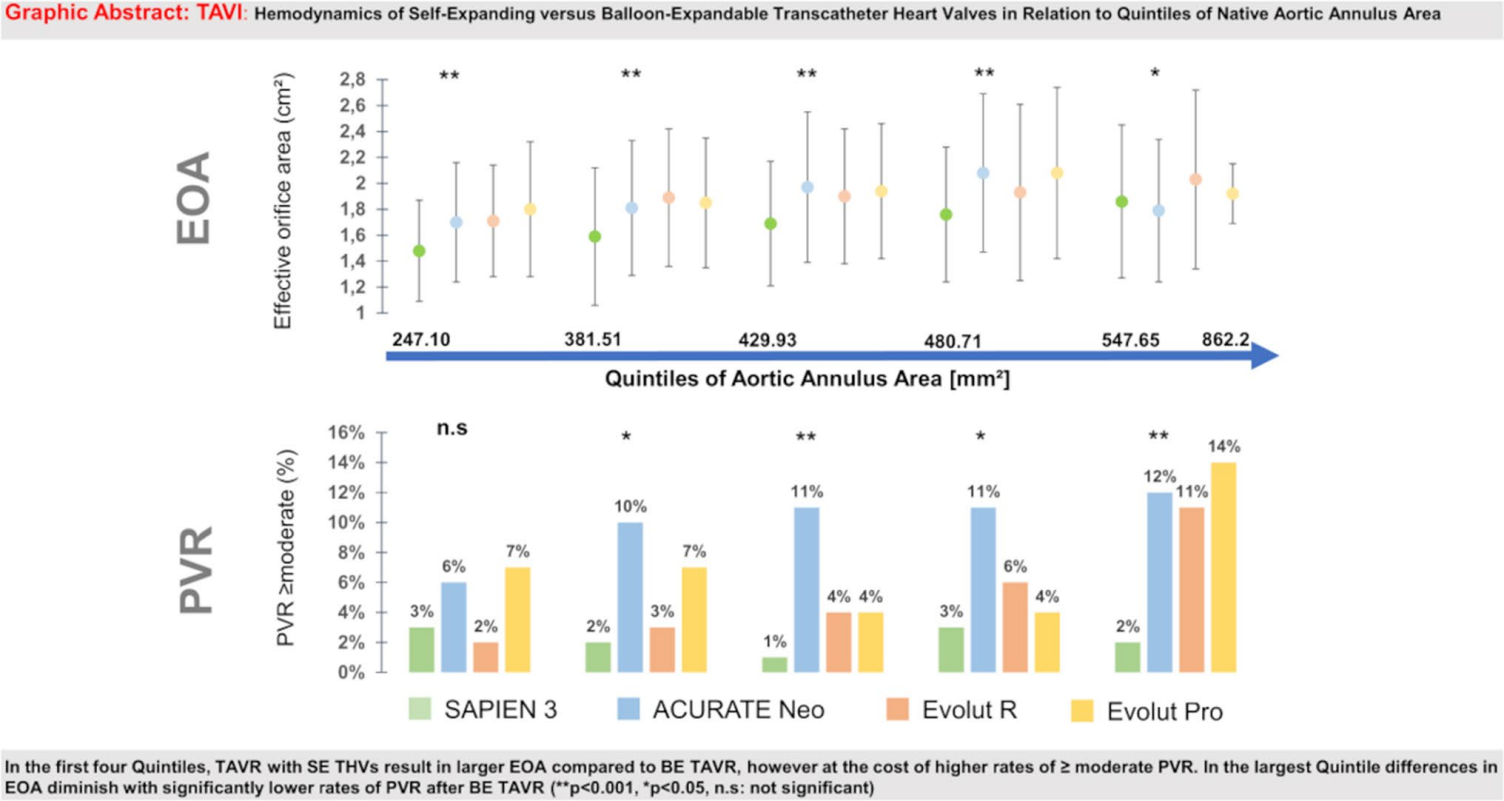

## Introduction

Transcatheter aortic valve replacement (TAVR) has emerged as a standard procedure for the treatment of severe aortic stenosis. Initially only performed in inoperable patients or at high perioperative risk [1, 2] subsequent randomized trials also confirmed safety and efficacy of TAVR in intermediate and low risk populations [3–5].

Currently, several balloon expandable (BE) or self-expanding (SE) transcatheter heart valves (THV) are available in clinical practice and distinct properties regarding hemodynamics have previously been outlined. Thereby, SE THV tend to have lower post-procedural mean transvalvular gradients (MG), larger effective orifice areas (EOA), and subsequently lower rates of severe patient-prosthesis mismatch (PPM). On the contrary, SE devices showed higher rates of relevant paravalvular regurgitation (PVR) compared to BE devices [6–9].

As TAVR indications extend towards younger patients at lower risk, hemodynamic characteristics of different TAVR protheses became a primary focus of clinical attention. However, comparative data of different valve types are rare. Importantly, sizing algorithms vary substantially between different valve types making it impossible to compare different valves of the same label size. For instance, a SAPIEN 3 26 mm valve covers a substantially different range of native annulus anatomy compared to a 26 mm Evolut R/Pro or ACURATE Neo M device [10]. Consequently, expectable hemodynamic characteristics of different THVs in relation to the dimension of a patient’s native aortic annulus would be utterly useful in prosthesis selection. In order to overcome these sizing algorithms, we herein report a comprehensive analysis of echocardiographic hemodynamic characteristics of three SE (Evolut R/Pro; ACURATE neo) and one BE (SAPIEN 3) THV with regard to native aortic annulus area obtained from a large multi-center real-world population. We also report echocardiographic data on different valve types and label sizes.

## Methods

2609 patients receiving TAVR at three high-volume sites in Germany were included into the analysis. TAVR was performed with either the balloon-expandable SAPIEN 3 (S3) THV (Edwards Lifescience, Irvine CA, USA; *n* = 1146) or self-expanding devices including the ACURATE neo (Neo) (Boston Scientific, Marlborough MA, USA; *n* = 649), Evolut R (ER) (Medtronic, Minneapolis MN, USA; *n* = 546) and Evolut Pro (Pro) (Medtronic, Minneapolis MN, USA; *n* = 268). At least three different label sizes were included for the comparison of S3, ER, Neo devices (23, 26, 29 mm; S, M, L) albeit the Pro 23 mm THV was excluded, due to the small number of patients treated with 23 mm THVs in our cohort (*n* = 2). Inclusion criteria were successful TAVR with one of the dedicated valves, the integrity of pre-procedural CT measurements as well as availability of post-intervention echocardiography. Patients undergoing valve-in-valve TAVR were excluded. There were no inclusion or exclusion criteria regarding patient characteristics. Prosthesis selection was at the discretion of the local heart team at each site. All patients provided written informed consent for intervention and data acquisition. The study was approved by the ethics committee of the University of Cologne (ID 19-1032) and has been conducted in accordance with the Declaration of Helsinki. Clinical and procedural data as well as baseline criteria for each patient were collected and compiled in a dedicated pseudonymized database.

### Pre-procedural MSCT analysis

**Fig. 1:**
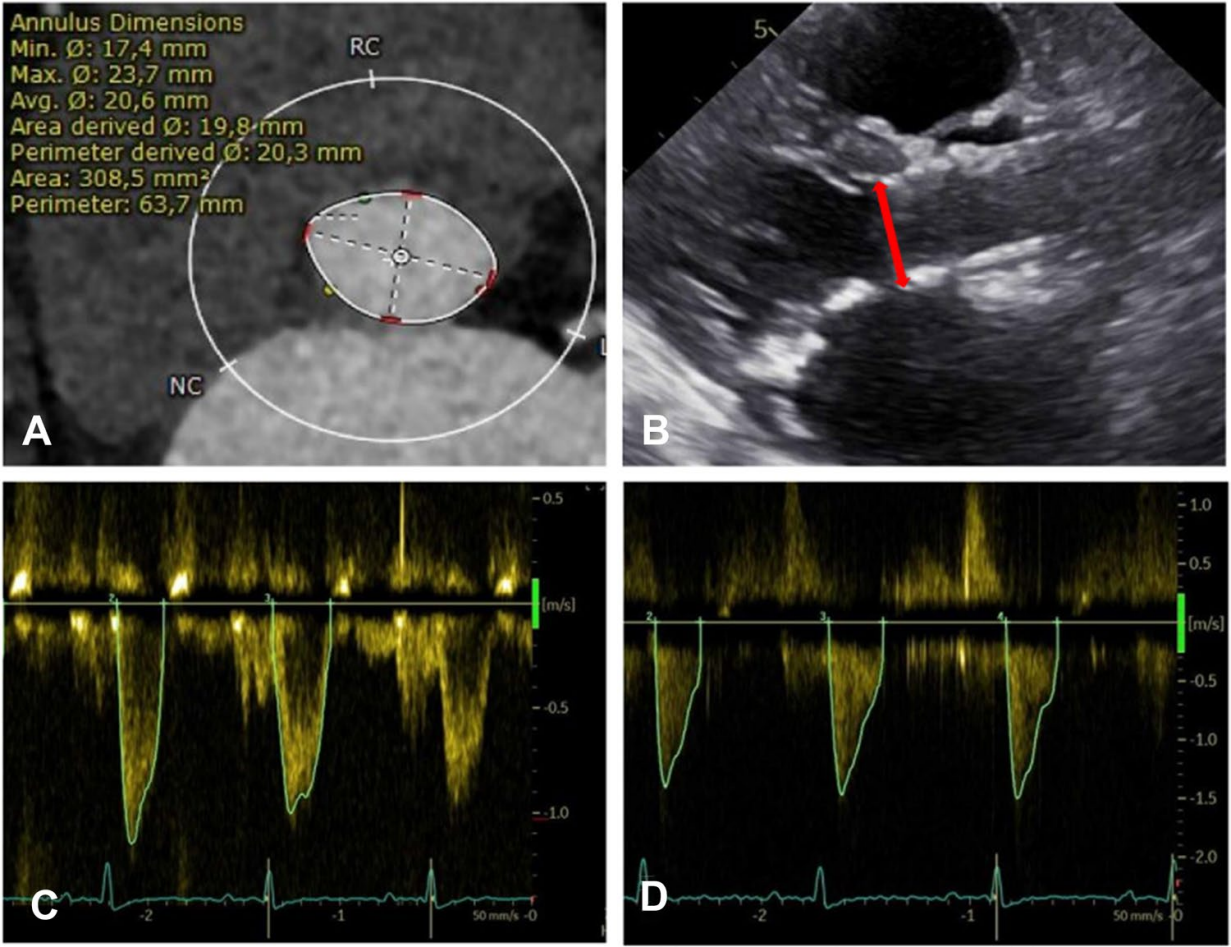
Multisliced Computed Tomography and Echocardiographic Measurements. **A** The Aortic Annulus was measured at the nadirs of the coronary cusps. **B** The left ventricular outflow tract (LVOT) (red line), used for the calculation of effective orifice area, was measured from the outer-to-outer border of the ventricular side of the stented valve. **C** Continuous wave doppler was measured across the aortic valve. **D** A pulsed-wave Doppler sample was placed in the LVOT to measure the LVOT velocity time integral

Each patient underwent pre-procedural contrast-enhanced multisliced computed tomography using standard methodology [11]. The aortic annulus plane was defined as a virtual ring at the nadirs of the three aortic valve cusps (Fig. 1A). All CT evaluations were performed with 3mensio Structural Heart 10.0 SP1.

### Post-procedural echocardiography

Patients underwent post-procedural transthoracic echocardiography before discharge (Fig. 1B–D). Mean gradients (MG) across the prosthetic valve were quantified by continuous wave (CW) Doppler. In patients with atrial fibrillation, gradients were calculated as the mean of five consecutive heart beats [12]. The neo-left ventricular outflow tract (LVOT) diameter was measured from the outer-to-outer border of the ventricular side of the stented valve, ideally in parasternal long axis view. If measurements of the outer-to-outer border were not possible, in stent measurements were performed. LVOT Velocity Time Integral (VTI) was measured with a pulsed-wave (PW) Doppler sample volume placed at a corresponding LVOT position. Doppler velocity index (DVI) was calculated as the ratio of PW Doppler LVOT VTI to CW Doppler derived aortic valve VTI. The effective orifice area (EOA) was computed with the continuity equation. EOA was then indexed to body surface area as calculated by the DuBois formula. According to VARC-2 recommendations, severe PPM was defined as EOAi < 0,65 cm^2^/m^2^ and < 0,60 cm^2^/m^2^ in obese patients (BMI ≥ 30 kg/m^2^) [13]. PVR was assessed using a multiparametric approach following current guidelines, and for statistical analysis categories were collapsed to < moderate or ≥ moderate [13].

### Statistics

Continuous parameters are reported as mean ± SD, whereas categorial variables are reported as frequencies and percentages. Data was tested for normal distribution using the Kolmogorow- Smirnow Test. Subsequently, differences of continuous and categorical variables were tested with the Kruskal–Wallis-Test, Fisher’s Exact Test or Chi Square test, as applicable. Post-hoc comparisons were adjusted using Bonferroni correction for multiple testing. Two-sided *P*-Values < 0.05 were considered statistically significant. All statistical analyses were performed with IBM SPSS statistics version 27 IBM.

## Results

### Patient population and baseline characteristics

2609 patients with severe aortic stenosis underwent TAVR at the three sites in Germany with either the BE THV SAPIEN 3 (*n* = 1146) or SE devices including ACURATE neo (*n* = 649), Evolut R (*n* = 546) or Evolut Pro (*n* = 268), respectively. Baseline characteristics according to implanted valve type are listed in (Table 1). The prevalence of previous cardiac surgery (highest in S3 with 18.2%, lowest in Pro with 7.5%; *p* < 0.001) and atrial fibrillation (highest in ER with 43.2%, lowest in Pro with 31.2%; *p* = 0.008) differed significantly between valve types while other comorbidities were equally distributed. EuroScore II was higher in patients treated with ER/Pro devices (*p* < 0.001). Patients receiving TAVR with S3 were more frequently male and had larger annular dimensions (*p* < 0.001). Predilation was performed in 41.9, 91.8, 30.4, and 45.1% of S3, Neo, ER and Pro patients, respectively (*p* < 0.001). Postdilation was conducted in 12.4, 33.1, 26.6, 32.8% of patients treated with S3, Neo, ER or Pro (*p* < 0.001).

**Table 1:** Baseline patient characteristics by valve type

	SAPIEN 3 (*n* = 1146)	ACURATE NEO (*n* = 649)	Evolut R (*n* = 546)	Evolut Pro (*n* = 268)	*P*-value
Age (years)	80.8 ± 6.7	82.4 ± 5.4	82.1 ± 6.1	82.0 ± 5.6	** < 0.001**
Male	663 (57.9%)	190 (29.3%)	212 (38.8%)	104 (38.8%)	** < 0.001**
Female	483 (42.1%)	459 (70.7%)	334 (61.2%)	164 (61.2%)	
BMI [kg / m^2^]	27.0 ± 5.1	27.0 ± 4.9	26.8 ± 5.2	26.5 ± 5.0	0.215
Extracardiac arteriopathy	291 (25.4%)	128 (19.8%)	131 (24.0%)	59 (22.0%)	**0.050**
Diabetes	338 (29.5%)	192 (29.7%)	167 (30.6%)	67 (25.0%)	0.408
Hypertension	1018 (88.8%)	582 (90.0%)	490 (89.9%)	229 (85.4%)	0.208
Coronary artery disease	725 (63.4%)	387 (60.1%)	342 (62.6%)	152 (56.7%)	0.166
Previous cardiac surgery	209 (18.2%)	73 (11.3%)	97 (17.8%)	20 (7.5%)	** < 0.001**
Renal disease [GFR ≤ 60 ml]	676 (59.0%)	373 (57.5%)	340 (62.3%)	146 (54.5%)	0.153
Atrial fibrillation	429 (38.0%)	258 (40.2%)	234 (43.2%)	83 (31.2%)	**0.008**
EuroSCORE II [%]	3.7 ± 6.6	3.4 ± 4.5	5.7 ± 6.8	4.7 ± 4.3	** < 0.001**
Baseline echocardiographic parameters					
LVEF					** < 0.001**
> 50%	708 (62.2%)	453 (72.4%)	367 (68.6%)	195 (73.6%)	
41–50%	183 (16.1%)	80 (12.8%)	71 (13.3%)	35 (13.2%)	
31–40%	135 (11.9%)	63 (10.1%)	63 (11.8%)	24 (9.1%)	
< 30%	113 (9.9%)	30 (4.8%)	34 (6.4%)	11 (4.2%)	
Mean gradient [mmHg]	37.7 ± 18.7	34.4 ± 19.2	34.9 ± 20.7	48.1 ± 19.5	** < 0.00**1
CT Measurements					
Annulus area [mm^2^]	498.7 ± 99.6	433.9 ± 75.2	452.0 ± 96.4	431.2 ± 61.6	** < 0.001**
Annulus perimeter [mm^2^]	80.4 ± 8.0	75.1 ± 6.3	76.4 ± 8.1	75.0 ± 5.2	** < 0.001**
Area derived diameter [mm]	25.1 ± 2.5	23.4 ± 2.0	23.8 ± 2.5	23.4 ± 1.7	** < 0.001**

Values are presented as mean ± SD or *n* (%).

Significant values are presented in bold letters

### Post-procedural hemodynamics based on valve type and label size

**Fig. 2:**
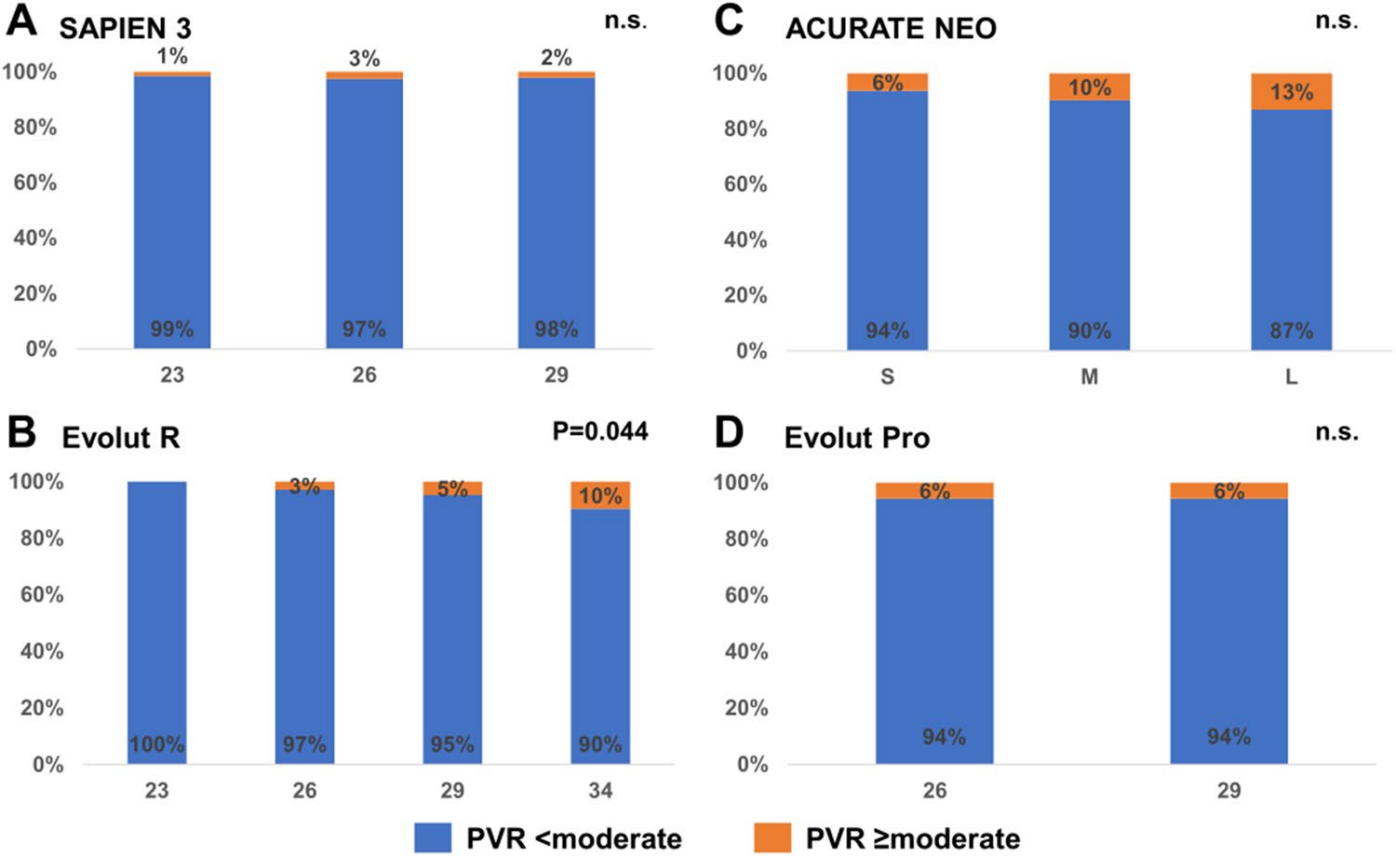
Paravalvular regurgitation ≥ moderate by Valve Type and Label Size. PVR ≥ moderate is rare in patients treated with **A** SAPIEN 3 valves (1, 3, 2%; *p* = 0. 636). PVR rises significantly with increasing valve size for **B** Evolut R devices (0, 3, 5, 10%; *p* = 0.044) and in different sizes of **C** ACURATE neo valves (6, 10, 13%; *p* = 0.090) without reaching statistical significance. PVR is identical in **D** 26, 29 mm Evolut Pro devices (6%; *p* = 1.0) (*n.s* not significant, *PVR* paravalvular regurgitation)

Tables 2, 3, 4 present the EOA, EOAi, DVI, MG, incidence of PVR ≥ moderate and > severe PPM for the different sizes of S3 (23, 26, 29 mm), Neo (S, M, L), ER (23, 26, 29, 34 mm) and Pro (23, 26 mm) THVs, respectively. For all valves, EOA and EOAi increased significantly with rising label valve size while mean gradients decreased. The incidence of PVR ≥ moderate was low in S3 THV without differences between valve sizes (1, 3, and 2% for 23, 26, and 29 mm valves, respectively; *p* = 0.636). In Neo devices, the rate of PVR ≥ moderate increased numerically with increasing valve sizes without reaching statistical significance (6, 10, 13% for S, M, L valves, respectively; *p* = 0.090). Relevant PVR differed significantly in ER treated patients depending on valve size, with more patients exhibiting PVR ≥ moderate with increasing label valve size (0, 3, 5, 10% for ER 23, 26, 29 and 34 mm, respectively; *p* = 0.044). The rate of relevant PVR was identical for 26 and 29 mm Pro THV (6%, *p* = 1.00; Fig. 2A–D). Rates of severe PPM were the highest in SAPIEN 3 THVs (12% *p* < 0.001) and lowest in NEO devices (3.7% *p* = 0.352).

**Table 2:** EOA, iEOA, DVI and MG for Balloon-Expandable SAPIEN 3

	23	26	29	All sizes	*P* value
	(*n* = 277)	(*n* = 481)	(*n* = 388)	(*n* = 1146)	
Effective orifice area [cm^2^]	1.47 ± 0.45	1.74 ± 0.50	1.86 ± 0.58	1.72 ± 0.54	** < 0.001**
Effective orifice area index [cm^2^/m^2^]	0.86 ± 0.29	0.93 ± 0.28	0.94 ± 0.31	0.92 ± 0.29	** < 0.001**
Doppler velocity index	0.50 ± 0.13	0.52 ± 0.13	0.50 ± 0.15	0.51 ± 0.14	**0.002**
Mean gradient [mmHg]	12.8 ± 5.0	11.0 ± 4.3	10.2 ± 4.1	11.2 ± 4.5	** < 0.001**
Paravalvular regurgitation ≥ moderate [%]	4 (1.5%)	12 (2.5%)	8 (2.1%)	24 (2.1%)	0.636
Severe PPM	48(17.3%)	40 (8.3%)	49(12.6%)	137 (12%)	< 0.001

Values are presented as mean ± SD or n (%)

Significant values are presented in bold letters

**Table 3:** EOA, iEOA, DVI and MG for Self-expanding ACURATE Neo

	S	M	L	All sizes	*P* value
	(*n* = 211)	(*n* = 282)	(*n* = 156)	(*n* = 649)	
Effective orifice area [cm^2^]	1.72 ± 0.49	1.85 ± 0.49	2.11 ± 0.66	1.87 ± 0.55	< 0.001
Effective orifice area index [cm^2^/m^2^]	1.01 ± 0.31	1.04 ± 0.28	1.11 ± 0.36	1.05 ± 0.31	0.007
Doppler velocity index	0.62 ± 0.15	0.63 ± 0.14	0.65 ± 0.14	0.63 ± 0.14	0.069
Mean gradient [mmHg]	9.1 ± 4.3	7.5 ± 3.5	6.6 ± 2.9	7.8 ± 3.8	< 0.001
Paravalvular regurgitation ≥ moderate [%]	13 (6.3%)	27 (9.6%)	20 (13.1%)	60 (9.4%)	0.090
Severe PPM	10 (4.7%)	7 (2.5%)	7 (4.5%)	24 (3.7%)	0.352

Values are presented as mean ± SD or *n* (%)

**Table 4:** EOA, iEOA, DVI and MG for Self-Expanding Evolut R / Pro

	23	26	29	34	All sizes	*P* value
	(*n* = 34)	(*n* = 174)	(*n* = 213)	(*n* = 125)	(*n* = 546)	
Evolut R effective orifice area [cm^2^]	1.42 ± 0.43	1.82 ± 0.44	1.87 ± 0.57	2.11 ± 0.68	1.88 ± 0.58	** < 0.001**
Effective orifice area index [cm^2^/m^2^]	0.84 ± 0.29	1.09 ± 0.30	1.04 ± 0.34	1.08 ± 0.36	1.05 ± 0.33	** < 0.001**
Doppler velocity index	0.57 ± 0.19	0.68 ± 0.15	0.62 ± 0.16	0.58 ± 0.17	0.63 ± 0.17	** < 0.001**
Mean gradient [mmHg]	12.3 ± 5.5	7.4 ± 3.8	7.7 ± 3.8	6.9 ± 4.1	7.7 ± 4.1	** < 0.001**
Paravalvular regurgitation ≥ moderate [%]	0 (0.0%)	5 (2.9%)	10 (4.7%)	12 (9.6%)	27 (5.0%)	**0.044**
Severe PPM	11 (32.4%)	3 (1.7%)	11 (5.2%)	7 (5.6%)	32 (5.9%)	** < 0.001**
Evolut Pro	*n* = 0	*n *= 89	*n* = 179	*n* = 0	*n* = 268	
Effective orifice area [cm^2^]	NA	1.75 ± 0.50	1.99 ± 0.55	NA	1.91 ± 0.54	** < 0.001**
Effective orifice area index [cm^2^/m^2^]	NA	1.03 ± 0.30	1.09 ± 0.32	NA	1.07 ± 0.31	0.307
Doppler velocity Index	NA	0.67 ± 0.16	0.65 ± 0.16	NA	0.66 ± 0.16	0.148
Mean gradient [mmHg]	NA	9.2 ± 5.7	7.9 ± 4.0	NA	8.4 ± 4.6	0.160
Paravalvular regurgitation ≥ moderate [%]	NA	5 (5.7%)	10 (5.6%)	NA	15 (5.7%)	1.00
Severe PPM	NA	10 (11.2%)	6 (3.4%)	NA	16 (6.0%)	0.010

Values are presented as mean ± SD or n (%)

Significant values are presented in bold letters

### Hemodynamics in relation to quintiles of native aortic annulus area

The study cohort was divided into quintiles based on CT-assessed native aortic annulus area (1st Quintile: 247.10–381.50 mm^2^; 2nd Quintile: 381.51–429.92 mm^2^; 3rd Quintile: 429.93–480.70 mm^2^; 4th Quintile: 480.71–547.64 mm^2^; 5th Quintile: 547.65–862.20 mm^2^). Table 5 presents the EOA, EOAi, DVI, MG, as well as rates of ≥ moderate PVR and severe PPM of the four different THVs over quintiles of native annular anatomy. With increasing annulus size, EOA rised significantly in S3 (*p* < 0.001), Neo (*p* < 0.001) and ER (*p* = 0.011) devices while MG decreased (*p* = 0.001; *p* < 0.001; *p* = 0.635 for S3, Neo, ER respectively). In Pro treated patients, EOA (*p* = 0.180) tended to rise simultaneously with aortic annulus area while DVI (*p* = 0.142) and MG (*p* = 0.744) decreased numerically without reaching statistical significance.

**Table 5:** Reference values for valve types by quintiles of native annulus area

	247.10 to 381.50 mm^2^	381.51 to 429.92 mm^2^	429.93 to 480.70 mm^2^	480.71 to 547.64 mm^2^	547.65 to 862.2 mm^2^	*P* values
SAPIEN 3	*n* = 148	*n* = 171	*n* = 203	*n* = 253	*n* = 371	
EOA [cm^2^]	1.48 ± 0.39	1.59 ± 0.53	1.69 ± 0.48	1.76 ± 0.52	1.86 ± 0.59	** < 0.001**
EOAi	0.88 ± 0.25	0.89 ± 0.33	0.92 ± 0.27	0.92 ± 0.28	0.94 ± 0.32	0.239
DVI	0.50 ± 0.12	0.51 ± 0.14	0.51 ± 0.13	0.51 ± 0.14	0.50 ± 0.15	0.104
Mean gradient	12.1 ± 4.9	12.0 ± 4.9	11.2 ± 4.4	10.8 ± 4.0	10.6 ± 4.5	**0.001**
PVR ≥ moderate	4 (2.8%)	4 (2.4%)	1 (0.5%)	7 (2.8%)	8 (2.2%)	0.387
Severe PPM	23 (15.5%)	26 (15.2%)	17 (8.4%)	28 (11.1%)	43 (11.6.0%)	**0.182**
ACURATE NEO	*n* = 178	*n* = 158	*n* = 143	*n* = 129	*n* = 41	
EOA [cm^2^]	1.70 ± 0.46	1.81 ± 0.52	1.97 ± 0.58	2.08 ± 0.61	1.79 ± 0.55	** < 0.001**
EOAi [cm^2^/m^2^]	0.99 ± 0.27	1.05 ± 0.32	1.09 ± 0.32	1.11 ± 0.34	0.95 ± 0.29	** < 0.001**
DVI	0.62 ± 0.15	0.63 ± 0.14	0.65 ± 0.16	0.64 ± 0.14	0.62 ± 0.13	0.485
Mean gradient	9.0 ± 3.7	8.4 ± 4.22	6.7 ± 3.6	6.6 ± 2.9	8.3 ± 3.1	** < 0.001**
PVR ≥ moderate	11 (6.4%)	15 (9.6%)	15 (10.6%)	14 (10.9%)	5 (12.2%)	0.515
Severe PPM	9 (5.1%)	8 (5.1%)	1(0.7%)	4 (3.1%)	2 (4.9%)	0.133
Evolut R	*n* = 136	*n* = 119	*n* = 97	*n* = 91	*n* = 103	
EOA [cm^2^]	1.71 ± 0.43	1.89 ± 0.53	1.90 ± 0.52	1.93 ± 0.68	2.03 ± 0.69	**0.011**
EOAi [cm^2^/m^2^]	1.04 ± 0.29	1.07 ± 0.33	1.06 ± 0.32	1.04 ± 0.38	1.05 ± 0.35	0.754
DVI	0.67 ± 0.16	0.67 ± 0.17	0.62 ± 0.16	0.58 ± 0.17	0.56 ± 0.16	** < 0.001**
Mean gradient	8.1 ± 4.6	7.9 ± 3.9	7.4 ± 3.7	7.3 ± 3.8	7.7 ± 4.6	0.635
PVR ≥ moderate	3 (2.2%)	4 (3.4%)	4 (4.2%)	5 (5.5%)	11 (10.8%)	0.058
Severe PPM	10 (7.4%)	7 (5.9%)	4 (4.1%)	8 (8.8%)	3 (2.9%)	0.390
Evolut Pro	*n* = 62	*n* = 72	*n* = 79	*n* = 48	*n* = 7	
EOA [cm^2^]	1.80 ± 0.52	1.85 ± 0.50	1.94 ± 0.52	2.08 ± 0.66	1.92 ± 0.23	0.180
EOAi [cm^2^/m^2^]	1.06 ± 0.32	1.06 ± 0.29	1.06 ± 0.30	1.11 ± 0.39	1.03 ± 0.11	1.0
DVI	0.69 ± 0.14	0.66 ± 0.17	0.64 ± 0.16	0.65 ± 0.17	0.57 ± 0.07	0.142
Mean gradient	9.0 ± 5.9	8.3 ± 4.0	8.5 ± 4.4	7.8 ± 4.2	6.6 ± 2.5	0.744
PVR ≥ moderate	4 (6.6%)	5 (7.0%)	3 (3.8%)	2 (4.2%)	1 (14.3%)	0.586
Severe PPM	6 (9.7%)	5 (6.9%)	4 (5.1%)	1 (2.1%)	0 (0%)	0.546
*P* values						
EOA [cm^2^]	< 0.001	< 0.001	< 0.001	< 0.001	0.034	
EOAi [cm^2^/m^2^]	< 0.001	< 0.001	< 0.001	< 0.001	0.017	
DVI	< 0.001	< 0.001	< 0.001	< 0.001	< 0.001	
Mean gradient	< 0.001	< 0.001	< 0.001	< 0.001	< 0.001	
PVR ≥ moderate	0.185	0.021	< 0.001	0.013	< 0.001	
Severe PPM	0.100	0.005	0.007	0.015	0.025	

Significant values are presented in bold letters

Values are mean ± SD or n (%). *P*-values are reported for comparisons between the different valve types within each quintile

^*^DVI doppler velocity index, †*EOA* effective orifice area, ‡*EOAi* effective orifice area indexed, §*PPM* patient-prosthesis mismatch, ∥ *PVR* paravalvular regurgitation

### Comparison of valve types by quintiles of native aortic annulus area

**Fig. 3:**
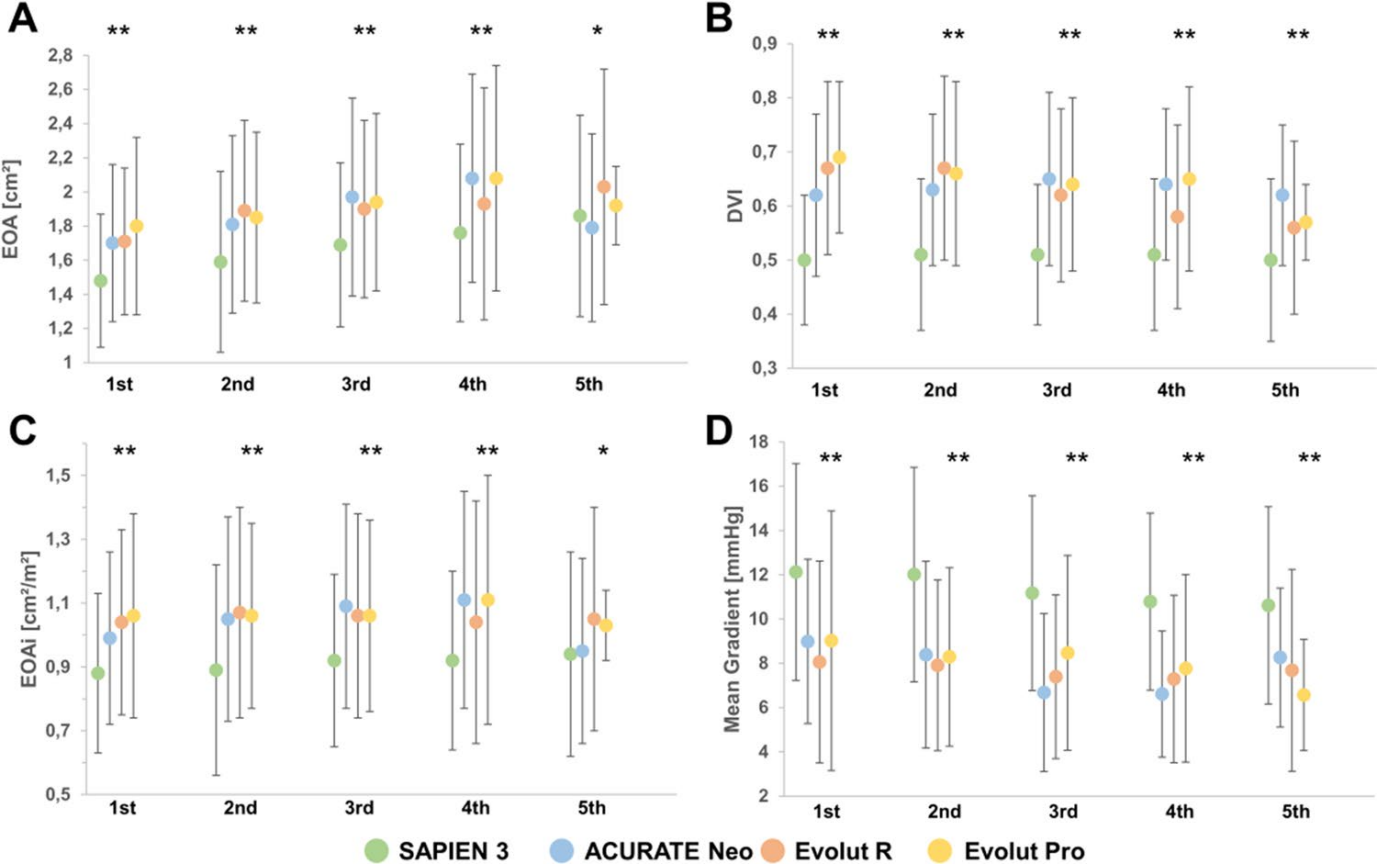
EOA, EOAi, DVI and Mean Gradient by Quintiles of Native Aortic Annulus Area (Central Illustration) EOA, EOAi are generally higher in SE compared to BE devices among the first four quintiles (*p* < 0.001). Differences between valve types are less pronounced in the largest quintile but still reach statistical significance (*p* = 0.034, *p* = 0.017 for EOA, EOAi). DVI is higher with SE compared to BE devices (*p* < 0.001), while MG (*p* < 0.001) are lower among all annular dimensions. (*DVI* doppler velocity index, *EOA* effective orifice area, *EOAi* effective orifice area index, *MG* mean gradient) (**p < 0.001, *p < 0.05)

**Fig. 4:**
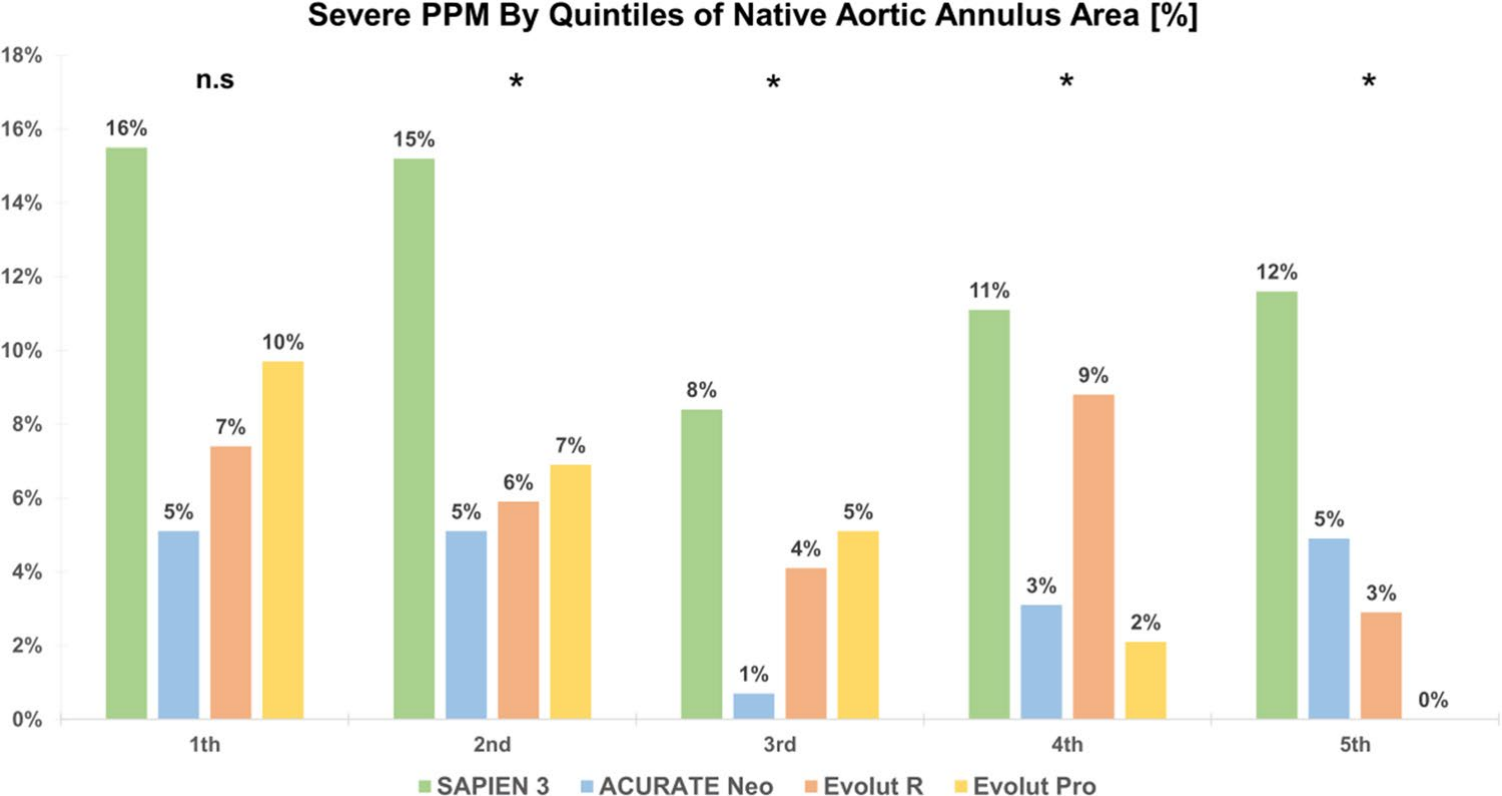
Severe Patient-Prosthesis Mismatch by Quintiles of Native Aortic Annulus Area. Among all Quintiles, severe PPM occurred less frequently in all self-expanding devices compared to S3 (*p* = 0.010, *p* = 0.005, *p* = 0.007, *p* = 0.015, *p* = 0.025). (*n.s* not significant, PPM = patient prosthesis mismatch), (**p < 0.001, *p < 0.05)

The hemodynamics of different valve types were compared within each quintile of native annular anatomy. Significant differences in terms of EOA/EOAi and MG between BE and SE THVs were observed with generally higher EOA/EOAi and lower MG for SE devices among different quintiles of annular dimensions (*p* < 0.05 for all comparisons: Table 5; Fig. 3). Severe PPM was subsequently more frequent in patients treated with S3 compared to SE THVs among all quintiles (Fig. 4).

DVI was significantly higher in SE than BE THV among all quintiles of annular dimensions (*p* < 0.001; Fig. 3B). The S3 presented with the highest post procedural MG (p < 0.001; Fig. 3D).

**Fig. 5:**
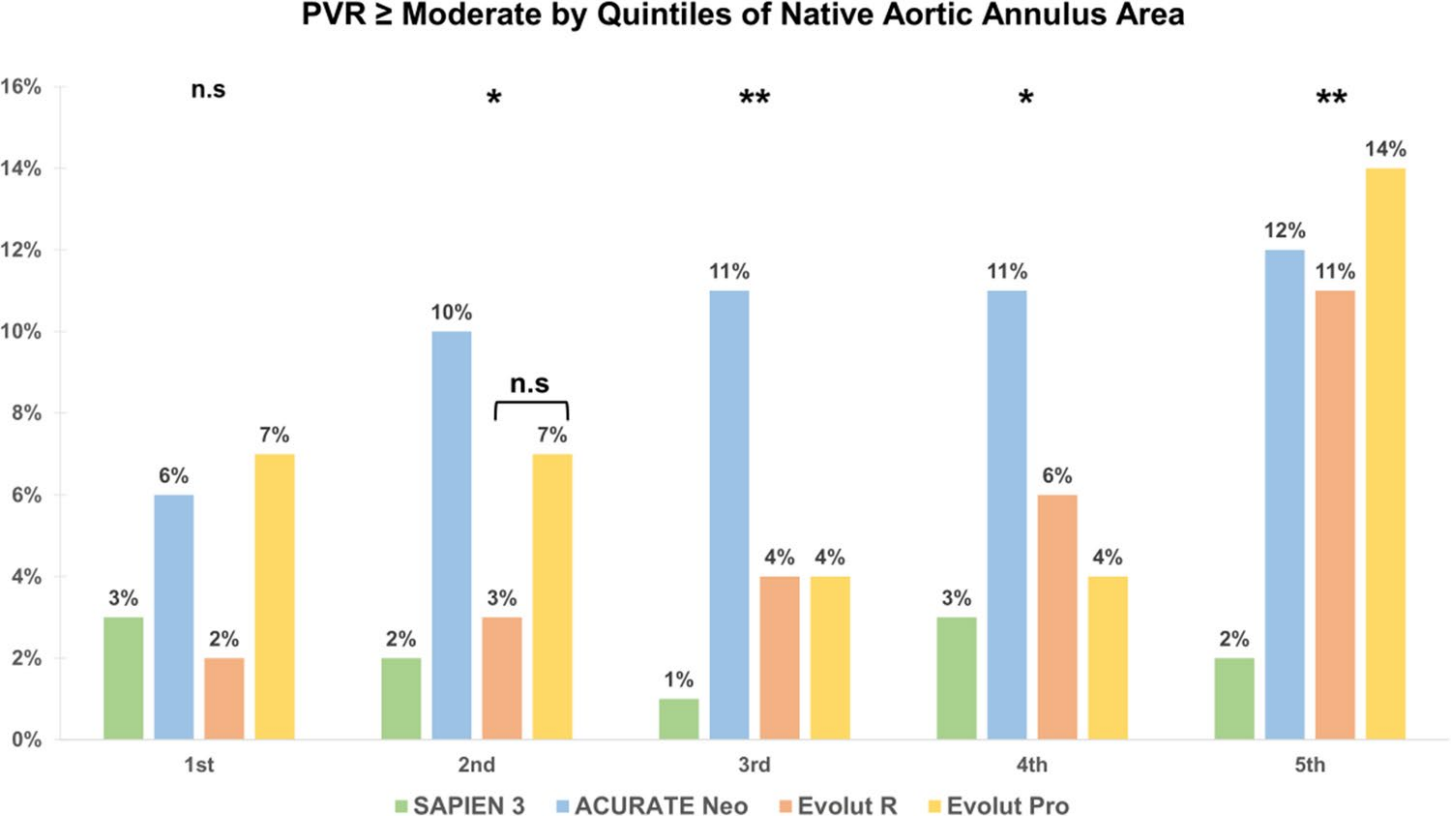
Paravalvular regurgitation ≥ moderate by Quintiles of Native Aortic Annulus Area. In the first quintile PVR ≥ moderate is similar in all THVs (*p* = 0.185). S3 presented with constantly low rates of ≥ moderate PVR whereas Neo averaged the highest rates of PVR ≥ moderate. ER/Pro presented with similar rates of relevant PVR with only slightly higher values than S3 THVs in the first four quintiles. In the largest quintile ≥ moderate PVR was significantly lower with S3 compared to SE devices (2 vs. 12 ,11, 14% *p* = 0.013) (*PVR* paravalvular regurgitation) (**p < 0.001, *p < 0.05)

In the first quintile, rates of ≥ moderate PVR were similar between all THVs (*p* = 0.185) while significant differences were observed in the other four quintiles. Thereby, the incidence of PVR ≥ moderate was lowest in S3 treated patients. In contrast, Neo devices showed the highest incidence of ≥ moderate PVR in the 2nd, 3rd and 4th quintiles while ER and Pro presented with similar occurrences. In the second quintile, Pro THV had a higher proportion of ≥ moderate PVR compared to ER (7 vs. 3%) albeit this difference was statistically not significant (*p* = 1.00). The lower rate of relevant PVR in S3 treated patients compared to SE THV treated patients was most pronounced in the largest quintile (> 547.65 mm^2^) (2 vs. 12, 11, 14% for S3 vs. Neo, ER, Pro respectively; *p* < 0.001, Fig. 5). Post-hoc analysis of different valve types confirmed that differences in EOA, EOAi, DVI and MG were largely driven by comparison of S3 THV compared to all SE devices while there were only few differences within the group of SEV THVs. Only in the fifth quintile (> 547.65mm^2^), there was no significant difference in EOA between SE and BE THVs (*p* > 0.05 for all comparisons). Rates ≥ moderate PVR were similar for ER and S3 in the lower four quintiles, while both THVs presented with significantly different rates in the fifth quintile (*p* < 0.05). The rate of relevant PVR was similar between NEO and S3 in the first quintile (*p* = 0.81) but not in larger annuli (all *p* < 0.05). ER and Pro THVs presented with similar rates of ≥ moderate PVR (all *p* > 0.05).

## Discussion

The present study represents the to date largest multicenter, real-world registry with comparative data of post-procedural hemodynamics of different SE and BE valve types both by valve type and label size, and also by quintiles of native annular dimensions. The main findings of our investigation are (1) SE THV provide superior hemodynamics in terms of larger EOA, EOAi, DVI, lower MG and lower rates of measured PPM in small annular dimensions, however, at the cost of higher rates of PVR ≥ moderate compared to BE THV. (2) Hemodynamic advantages seen with SE THV fade with increasing annulus size, whereas the rate of ≥ moderate PVR is significantly higher in patients treated with SE TAVR in large annuli (> 547.65mm^2^).

### Normative hemodynamic parameters by valve type and size

Reference data for expected post-procedural echo parameters by valve types and sizes are crucial in the evaluation of THV function. Previously, Hahn et al. proposed reference values for normal valve function of S3 and ER THV derived from the randomized PARTNER II trial and nested SAPIEN 3 registry and Core Valve Evolut R randomized trial, respectively [14]. The strength of that work is the high quality of core-lab assessed data, however derived from highly selected trial patients. In contrast to that controlled study population, we compiled non-randomized, real-world data based on an all-comers population without any predefined inclusion or exclusion criteria, reflecting real-world practice. Despite these differences, the assessed normative parameters showed high accordance in terms of EOA and MG for S3 THV. Smaller real-world registries have observed minor differences with higher EOA and lower MG [15, 16]. For ER and Neo, similar hemodynamics in terms of EOA and single-digit MG were reported in the Evolut R U.S. Study and the SAVI-TF registry, respectively [17, 18]. Hemodynamic data on the Evolut Pro is still limited yet comparable to our findings [19].

### Comparison of BE and SE THVs by quintiles of aortic annulus area

We compared the hemodynamics of BE and SE devices among different quintiles of native annulus dimensions to account for differences in sizing algorithms. As expected, EOA, EOAi and DVI were significantly higher and MG lower with SE THV compared to BE THV over all quintiles. The different prosthesis design with intraannular (BE) versus supraannular (SE) positioning of valve leaflets may be accountable for the hemodynamic variations seen between SE and BE THV [6, 20, 21]. Interestingly, these differences were more pronounced in smaller quintiles and attenuated with increasing annulus size, as reported similarly in two small previous studies comparing SE and BE devices [15, 22].

As a consequence of lower MG and larger EOA, the incidence of severe PPM was significantly lower after SE TAVR over all quintiles, in line with recent studies [7, 23]. The impact of PPM on outcomes after TAVR has been of current debate. In this study, PPM was calculated using measured iEOA instead of the predicted iEOA derived from reference values of normal valve function. There is an ongoing debate about the value of measured vs. predicted PPM with so far inconclusive results. After SAVR, predicted severe PPM has been associated with recurrence of heart failure symptoms and higher mortality [24, 25]. However, its consequence after TAVR remains controversial [26–28]. Recently published work confirmed the association of PPM and mortality after SAVR, but did not show such association after both BE and SE TAVR [29, 30]. Nevertheless, preventing PPM by larger EOA may be of high importance as the rates of PVR fall with new iterations of THVs and TAVR indications extend towards younger and healthier patients with a presumably longer life expectancy and higher level of activity.

PVR ≥ moderate has been associated with impaired outcomes post TAVR [31, 32] and defines unsuccessful device implantation in VARC-2. Consequently, the prevention of relevant PVR has been a major issue over the last years [9, 20, 21], leading to the development of dedicated sealing-mechanisms. In our study, SE valves presented with higher rates of ≥ moderate PVR compared to the S3 (S3: 2.1%; ER: 5.0%; Pro: 5.7%), with highest rates after TAVR with Neo (9.4%). For Neo, similar rates of PVR ≥ moderate have been recently described in the SCOPE 2 trial (9.6%) [33], whereas real-world studies showed substantially lower rates [6, 18]. Varying rates of ≥ moderate PVR have been reported within the literature, with a trend towards lower rates with newer generations of SE valves compared to previous generations [19, 34]. The difference in PVR rates was most pronounced in patients within the highest quintile of annulus area with rates of relevant PVR > 10% in all SE prostheses (S3: 2 vs. 12, 11, 14% for NEO, ER, Pro respectively; *p* < 0.001). However, results of the recent CHOICE Extend Registry indicate improved sealing mechanisms in SE THVs as there was no significant difference in any PVR between S3 and ER THVs in large and small annuli [35]. Rates of PVR were comparable between ER and Pro with a numerical trend to higher rates after Pro TAVI, most probably due to an inherent selection bias choosing a Pro valve in patients with higher calcium burden and thus higher anticipated PVR risk.

Based on the data of this study, SE TAVR may provide superior hemodynamics in terms of lower MG and consequently larger EOA in small and intermediate annular dimension at the cost of higher PVR rates. Whether improvements in valve design like the recent introduction of the Evolut Pro plus 34 mm or Accurate neo 2 may overcome the higher rate of relevant PVR in larger annular dimensions remains to be seen.

### Limitations

Since this is a retrospective multicenter analysis, typical limitations apply. We included an all-comers population, and the choice of THV was at the discretion of each local heart team. Thus, confounding variables that may influence THV hemodynamics cannot be excluded and must be taken into consideration. For instance, we cannot provide information on degree and distribution of aortic valve calcification. Moreover, hemodynamic echo parameters were site assessed, lacking a central core lab. Recent investigations proposed a potential discordance between invasive and echocardiographic transvalvular gradients post TAVR, especially in small BE THVs which have been reported to have lower mean gradients when measured invasively due to the phenomenon of pressure recovery [36]. However, post TAVR echocardiography remains the most common and practical method in the evaluation of post TAVR THV functioning and the role of invasive measurements remains to be defined. Finally, patient numbers and characteristics vary within the different groups and quintiles which may have influenced the observed results. We could not analyze data on 23 mm Pro devices owing to the small study cohort. Also, the presented post-hoc analyzes may lack appropriate statistical power to derive relevant conclusions.

## Conclusions

Our results suggest that TAVR with SE rather than BE devices may be beneficial in patients with small aortic annular dimensions as they provide larger EOAs and EOAis. Subsequently, severe PPM was less frequent with SE devices. MG were lower with SE compared to BE THVs while nearly comparable rates of ≥ moderate PVR were achieved. In contrast, it may be reasonable to select either valve type with increasing annulus sizes as differences in hemodynamics attenuate. In fact, it may even be preferable to implant BE devices in patients with extremely large annuli (> 547.64 mm^2^) as the occurrence of relevant PVR is significantly lower. The herein risen hemodynamic data may also be extremely helpful in the evaluation of long-term valve function and the early evaluation of structural valve deterioration.
